# Intelligence is associated with the modular structure of intrinsic brain networks

**DOI:** 10.1038/s41598-017-15795-7

**Published:** 2017-11-22

**Authors:** Kirsten Hilger, Matthias Ekman, Christian J. Fiebach, Ulrike Basten

**Affiliations:** 1 Goethe University Frankfurt, Frankfurt am Main, Germany; 2 IDeA Center for Individual Development and Adaptive Education, Frankfurt am Main, Germany; 3 Donders Institute for Brain, Cognition, and Behaviour, Radboud University, Nijmegen, The Netherlands

## Abstract

General intelligence is a psychological construct that captures in a single metric the overall level of behavioural and cognitive performance in an individual. While previous research has attempted to localise intelligence in circumscribed brain regions, more recent work focuses on functional interactions between regions. However, even though brain networks are characterised by substantial modularity, it is unclear whether and how the brain’s modular organisation is associated with general intelligence. Modelling subject-specific brain network graphs from functional MRI resting-state data (*N* = 309), we found that intelligence was not associated with global modularity features (e.g., number or size of modules) or the whole-brain proportions of different node types (e.g., *connector hubs* or *provincial hubs*). In contrast, we observed characteristic associations between intelligence and node-specific measures of within- and between-module connectivity, particularly in frontal and parietal brain regions that have previously been linked to intelligence. We propose that the connectivity profile of these regions may shape intelligence-relevant aspects of information processing. Our data demonstrate that not only region-specific differences in brain structure and function, but also the network-topological embedding of fronto-parietal as well as other cortical and subcortical brain regions is related to individual differences in higher cognitive abilities, i.e., intelligence.

## Introduction

Human intelligence describes the general mental capability that involves the ability to reason, to think abstractly, and to learn quickly from experiences^1^. It is associated with many important outcomes in life, including education, occupation, socioeconomic status^1^, health, and longevity^2^. Understanding the brain bases of human intelligence is one of the central goals of cognitive neuroscience. Previous brain imaging studies have linked general intelligence to the structure and function of frontal and parietal brain regions^3,4^. More recent work extends this localisationist approach and points to the importance of interactions within and between functional brain networks for explaining individual differences in general intelligence^5,6^.

The intrinsic connectivity of the human brain, i.e., its interregional coupling profiles during an alert but task-free state of rest, is assumed to reflect fundamental organisational principles of brain networks^7^. Intrinsic functional networks are closely related to underlying anatomical connections^8^, constrain brain activity during cognitive demands^9,10^, and are associated with fundamental differences between persons, e.g., in personality^11^ or psychopathology^12^.

Graph theory, a computational approach for the detailed modelling and characterisation of large-scale networks^13^, can be used to describe both the brain network as a whole as well as the connectivity profile of specific nodes within that network. To model the brain network as a graph, the brain is spatially parcellated into a set of regions that serve as network nodes. When functional networks are modeled, edges, i.e., functional connections, are defined between nodes with highly correlated time series of the blood-oxygen-level dependent (BOLD) signal. Together, the nodes and edges define a graph with a specific topology, whose functional properties can be described by various graph-theoretical metrics^13,14^. Investigations of intelligence-related differences in the topological organisation of brain networks have so far focused on the graph-theoretical concept of network efficiency and initially suggested an overall more efficient network topology in more intelligent persons due to on average shorter paths from any node in the network to any other^5^. However, in node-specific analyses, the association between intelligence and measures of network efficiency was found to vary between brain regions^6^.

Graph-theoretical investigations of intelligence and brain network connectivity have so far not considered that functional connections are not uniformly distributed across the network, but clustered into subnetworks (modules, communities) that are densely connected internally but only weakly coupled with the rest of the network^15^. Modular network organisation is a general feature of complex biological systems^15^ and has been associated with functional specialisation^16^ as well as with robustness and adaptability of the network system^17^. Within these modular brain networks, each node is characterised by a specific profile of within- and between-module connectivity, which determines a node’s functional role in neural processing within and across different modules^18^, and allows to classify nodes into different node types (e.g., *connector hubs*, *provincial hubs*), whose relative quantities may influence the information flow within the whole network.

Previous studies have begun to link individual differences in the modular organisation of functional brain networks (assessed either during cognitive tasks or resting state) to differences in cognitive functions. However, these initial investigations restricted their focus to specific domains of cognition. For example, associations were observed between individual working memory performance^19–22^ (e.g., n-back tasks) and different aspects of modular brain network organisation, i.e., (a) whole-brain measures of modular network organisation^19,20^, (b) proportions of specific node types within the modular brain network (i.e., *connector hubs*, *provincial hubs*)^20,22^, and (c) a node-specific measure of between-module connectivity^21^. Considering that working memory performance is closely linked to general intelligence^23^, these studies strongly suggest that individual differences in the modular organisation of functional brain networks could also more generally be relevant for higher cognitive ability, i.e., intelligence.

To explore the association between brain network modularity and general intelligence, we apply graph analyses to fMRI resting-state data and characterise the modular brain network organisation in a large and representative sample of healthy adults (*N* = 309). Specifically, we address the following research questions: (a) Are individual differences in general intelligence associated with differences in the overall modular organisation of the brain (e.g., *global modularity*, *number of modules*) or with differences in the proportions of node types (e.g., *connector hubs*, *provincial hubs*)? (b) Are node-specific aspects of modular organisation in circumscribed regions of the brain, i.e., a node’s between-module connectivity and within-module connectivity, related to differences in general intelligence?

## Methods

### Participants

The data used for this study were acquired by the Nathan Kline S. Institute for Psychiatric Research and made available by the 1000 Functional Connectomes Project INDI (Enhanced NKI Rockland Sample^24^, http://fcon_1000.projects.nitrc.org/indi/enhanced/). Institutional review board approval for this project was obtained at the Nathan Kline Institute (#239708). All methods were carried out in accordance with these guidelines and all participants gave written informed consent. We used a subsample of 309 participants, for which complete neuroimaging data were available (age: 18–60 years, *M* = 38.93, *SD* = 13.94; gender: 199 female, 110 male; handedness: 262 right, 22 left, 25 ambidextrous). For this sample, the Full Scale Intelligence Quotient (FSIQ), as assessed with the Wechsler Abbreviated Scale of Intelligence (WASI)^25^, ranged from 67 to 135 (*M* = 99.12, *SD* = 13.23).

### fMRI Data Acquisition

MRI data were acquired on a 3 Tesla whole-body MRI scanner (MAGNETOM Trio Tim, Siemens, Erlangen, Germany). Functional resting-state data were obtained using a T2*-weighted BOLD-sensitive gradient-echo EPI sequence with 38 transversal axial slices of 3 mm thickness (120 volumes; field of view [FOV] 216 × 216 mm; repetition time [TR] 2500 ms; echo time [TE] 30 ms; flip angle 80°; voxel size 3 × 3 × 3 mm; acquisition time 5.05 min). For coregistration, three-dimensional high-resolution anatomical scans were obtained with a sagittal T1-weighted, Magnetization Prepared-Rapid Gradient Echo sequence scan (176 sagittal slices; FOV 250 × 250 mm; TR 1900 ms; TE 2.5 ms; flip angle 9°; voxel size 1 × 1 × 1 mm; acquisition time 4.18 min).

### Preprocessing

Data was preprocessed using FSL (http://www.fmrib.ox.ac.uk/fsl/) and AFNI (http://afni.nimh.nih.gov/afni) with the scripts released by the 1000 Functional Connectomes Project (http://www.nitrc.org/projects/fcon_1000), comprising: 1. Discarding the first four EPI volumes to allow for signal equilibration, 2. Slice-time correction, 3. Three-dimensional motion correction, 4. Time-series despiking, 5. Spatial smoothing (6 mm full-width half-maximum Gaussian kernel), 6. Four-dimensional mean-based intensity normalisation, 7. Bandpass temporal filtering (0.005–0.1 Hz), 8. Removing linear and quadratic trends, 9. Normalisation of the individual EPI volumes to MNI152 space (3 × 3 × 3 mm) via nonlinear transformation and by the use of each subject’s anatomical scan, 10. Elimination of nine nuisance signals (white matter, cerebrospinal fluid, global mean, six motion parameters) by regression.

### Graph Analyses

Graph analyses were performed with the open source python package *network-tools* ^26^, specifically developed for the analysis of functional and structural brain network graphs.

#### Graph construction

As nodes, we used those 5,411 voxels that covered all grey matter in the EPI images down-sampled to 6 × 6 × 6 mm. For each subject separately, edges were assumed between nodes showing high positive correlations of BOLD signal time series. Edges of physically short distance (< 20 mm) were excluded, due to their increased susceptibility to motion artefacts and potential correlations arising from shared nonbiological signal^27^. Most graph metrics are strongly influenced by the density of the graph^28^. This has specifically been shown for modularity^29^. To avoid biases due to individual differences in graph density, the main analyses were performed on thresholded and binarised graphs (as recommended for the study of individual differences in graph topology^28,30^). In contrast, weighted graphs usually vary in density (i.e., the mean weight of edges), even if the number of edges is held constant across individuals. For the purpose of comparison, we also conducted all analyses on weighted graphs (see Supplementary Tables S4, S5). Discussion of results, however, will rely on the results for the binarised graphs. We applied five different thresholds to the correlation matrix, retaining the strongest 10, 15, 20, 25, or 30% edges, thereby also excluding all negative edges^31^. This resulted in five graphs of different density per person. Community detection and the calculation of graph metrics were performed separately for the five graphs, and resulting graph metrics were averaged for each participant. This averaging procedure was applied to enhance the reliability of findings, as the resulting measures of graph properties are robust across a wider range of thresholds.

#### Measures of modular network organisation

To study the modular organisation of the functional brain network graphs, we applied the Louvain algorithm^32^. It finds the optimal modular partition in an iterative procedure maximizing the *global modularity Q* ^33^:

1 $$Q={\sum }_{s=1}^{m}[\frac{{l}_{ins}}{L}-\,{(\frac{{k}_{s}}{2L})}^{2}]\,,$$

where *m* is the number of modules, *l*_*ins*_ is the number of edges inside module *s*, *L* is the total number of edges in the network, and *k*_*s*_ is the total degree of the nodes in module *s*. Thus, the actual fraction of within-module edges is represented by the first term, whereas the expected fraction of within-module edges is represented by the second term. If the first term (actual within-module edges) is much larger than the second term (expected within-module edges), there are many more edges inside module *s* than expected by chance. In that case, *s* can be defined as a module and the *global modularity Q*, which results from summing up these differences (actual – expected within-module edges) over all modules *m* in the network, is increased. Usually, modularity values of *Q* > 0.3 indicate a modular network structure^34^.

The Louvain algorithm starts by assigning a different module to each node. Then, the first step is a greedy optimisation, where nodes adopt the modules of one of their neighbour nodes, if this reassignment increases the *global modularity Q* (see above). In the second step, a meta-network is built, whose nodes are the modules found in the first step. Both steps are repeated until no improvement in *global modularity Q* is possible and the optimal partition is found^35–37^. In addition to *global modularity Q*, for each participant, we calculated three further whole-brain measures of modular network organisation for the final module partition: *number of modules*, *average module size*, and the *variability in module size*.

The embedding of each node within the modular partition can be described by two graph-theoretical metrics:

(i) The *participation coefficient p*_*i*_ represents between-module connectivity and is defined as:

2 $${p}_{i}=1-\,{\sum }_{m{\epsilon }M}{(\frac{{k}_{i}(m)}{{k}_{i}})}^{2}\,,$$

where *k*_*i*_ is the degree of node *i* (i.e., the number of edges directly attached to node *i*) and *k*_*i*_ *(m*) is the subset of edges that connect node *i* to other nodes within the same module^13,38^. The *participation coefficient p*_*i*_ of a node is 0 when all of its edges are within its own module, and close to 1 when its edges are uniformly distributed among its own and other modules^38^.

(ii) The *within-module degree z*_*i*_ represents within-module connectivity and is defined as:

3 $${z}_{i}=\,\frac{{k}_{i}\,({m}_{i})-\,\overline{k\,}\,({m}_{i})}{{\sigma }^{k({m}_{i})}},$$

where *m*_*i*_ is the module of node *i*, *k*_*i*_ *(m*_*i*_ *)* is the within-module degree of node *i*, $\bar{k}$ (*m*_*i*_) and *σ* ^*k(mi)*^ are the mean and standard deviation of the within-module degree distribution of module *m*_*i*_^38^. Positive values indicate that a node is highly connected to nodes within its own module, whereas negative values indicate low levels of connectivity within the same module^15^.

**Figure 1:**
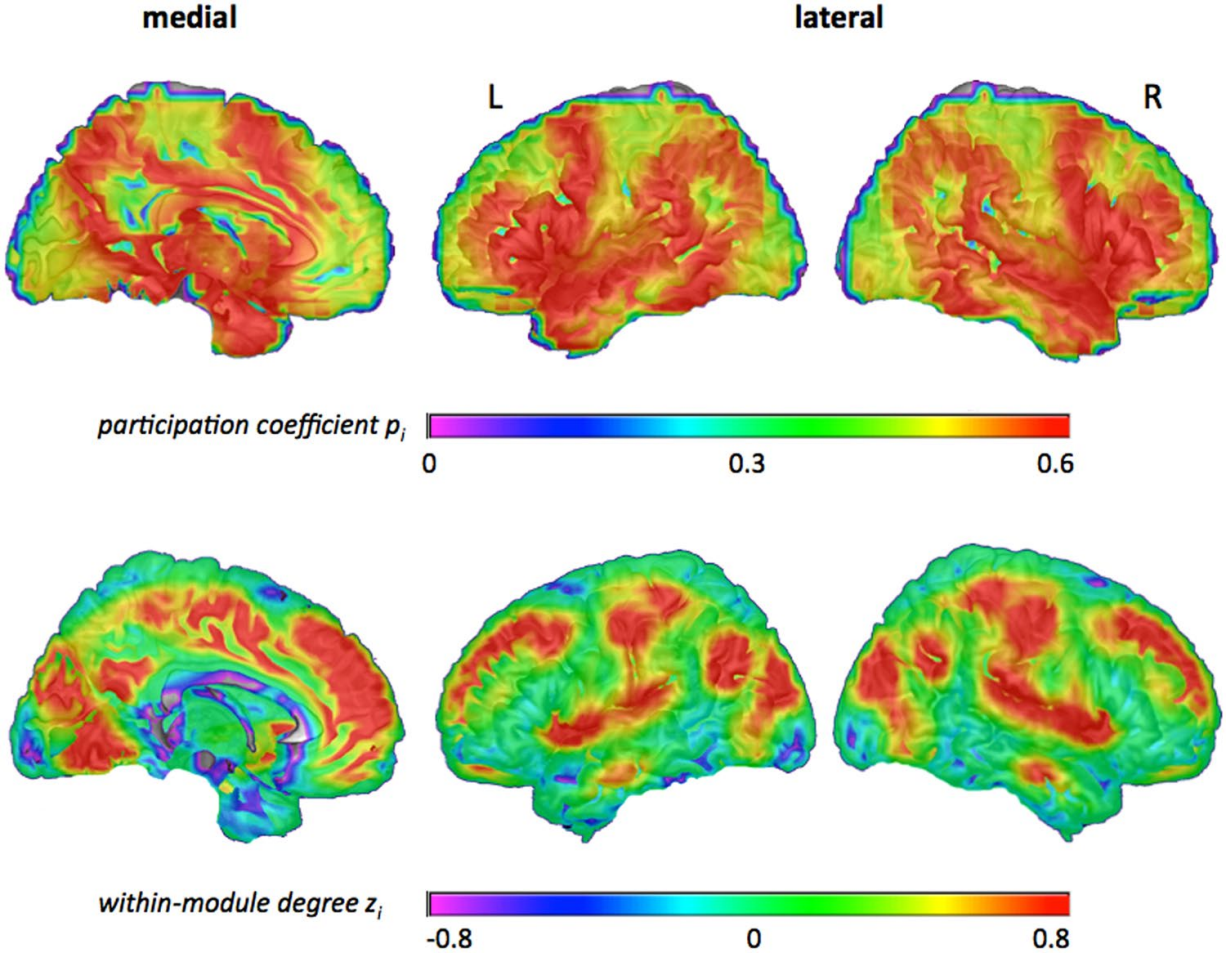
Values of *participation coefficient p*_*i*_ (top row) and *within-module degree z*_*i*_ (bottom row) averaged across all participants. Higher values are shown in warm colours (green to red), lower values are shown in cool colours (blue to pink). Graph metrics (*p*_*i*_ and *z*_*i*_) were calculated for binarised and proportionally thresholded graphs using five different cut-offs (i.e., graphs were defined by the 10%, 15%, 20%, 25%, or 30% strongest edges). For each participant, individual mean maps for the graph metrics were calculated by averaging across the five thresholds. Displayed here are the group average maps for *p*_*i*_ and *z*_*i*_ that resulted from averaging all individual mean maps across participants (see Methods section for details on the procedure). For the lateral view, values were projected to the surface of the brain. The medial view displays graph values in the *x*-plane. L, left; R, right.

These two graph metrics, *participation coefficient p*_*i*_ and *within-module degree z*_*i*_, allow the characterisation of a node’s embedding within the modular brain network free of any biases due to different module sizes^18^ (as would be the case when simply comparing numbers of between- and within-module connections). The distributions of *participation coefficient p*_*i*_ and *within-module degree z*_*i*_ were visualised by averaging the individual mean *p*_*i*_ *-* and *z*_*i*_ -values of each node across participants and projecting them to the surface of the brain (Fig. 1).

#### Node-type analysis

**Figure 2:**
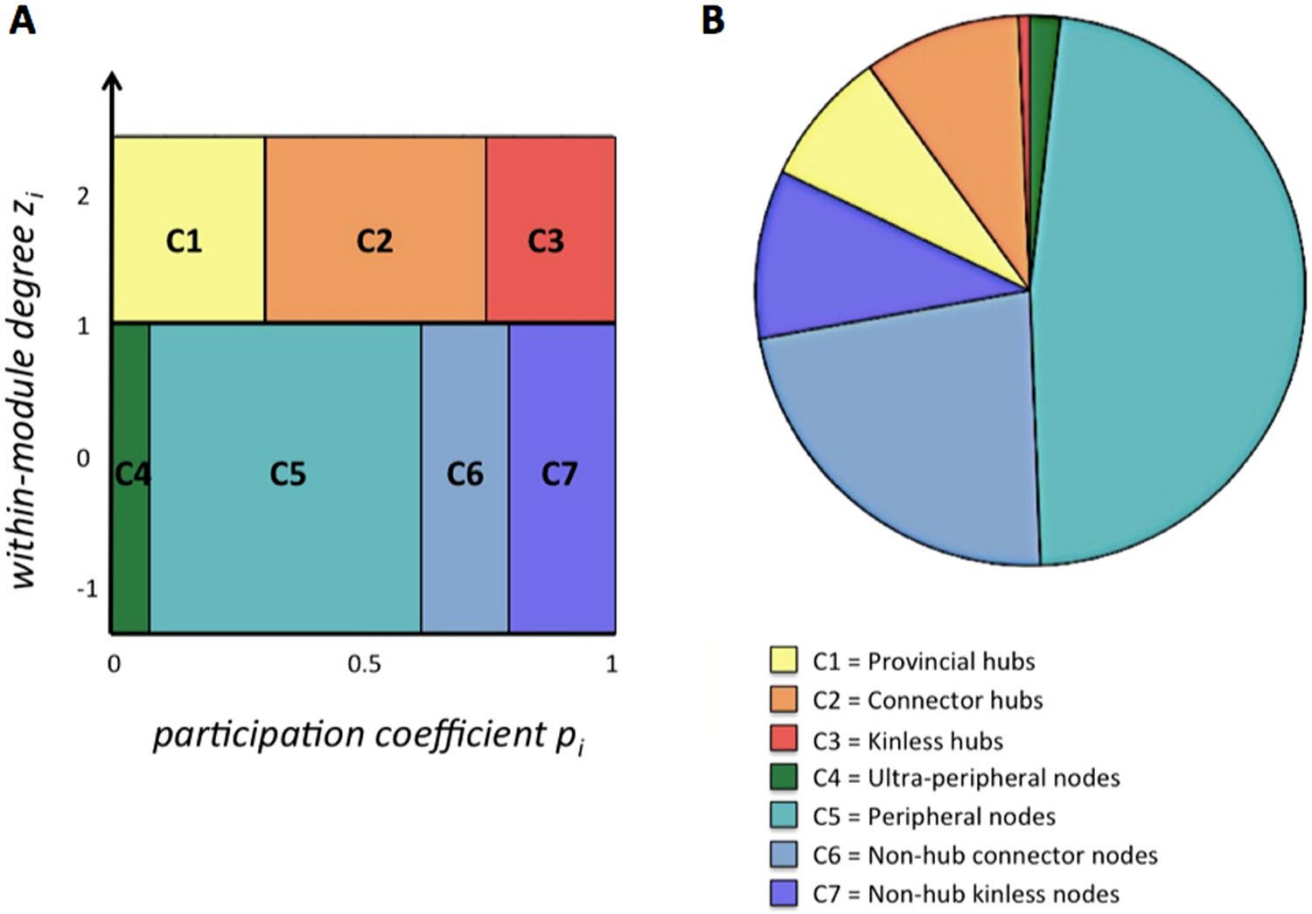
Node-type analysis. **(A)** Definition of node types as a function of *participation coefficient p*_*i*_ and *within-module degree z*_*i*_. Adapted from Guimerà and Amaral (2005; method also known as functional cartography; cf. Methods). **(B)** Group-average proportions of node types across the entire cortex. Proportions of node types were calculated for each individual subject separately and then averaged across all subjects. **(C)** Illustration and anatomical distribution of node types within the human brain for one exemplary participant. Non-hub nodes (*z*_*i*_ *≤ *1) are shown in cool colours (green to blue), hubs (*z*_*i*_ > 1) are shown in warm colours (yellow to red). Graph metrics (*p*_*i*_ and *z*_*i*_) and the respective node-type proportions were calculated for binarised and proportionally thresholded graphs using five different cut-offs (i.e., graphs were defined by the 10%, 15%, 20%, 25%, or 30% strongest edges). For each participant, individual node-type proportions were calculated by averaging across the five thresholds. Displayed in B are the group average proportions of node types resulted from averaging all individual node-type proportions across participants (see Methods section for details on the procedure). The *x*- and *z*-coordinates represent coordinates of the Montreal Neurological Institute template brain (MNI152).

Functional cartography^38^ uses the above-described metrics (*participation coefficient p*_*i*_, *within-module degree z*_*i*_) to classify network nodes into seven different types regarding their roles in within- and between-module communication (see Fig. 2A). As proposed in previous work^38,39^, we classified nodes with *within-module degree z*_*i  *_≥ 1 as hubs (18% of all nodes) and nodes with *z*_*i*_ < 1 as non-hubs. Depending on the *participation coefficient p*_*i*_, non-hubs were further divided into *ultra-peripheral* (*p*_*i*_ ≤ 0.05), *peripheral* (0.05*p*_*i*_ ≤ 0.62), *non-hub connector* (0.62 < *p*_*i*_ ≤ 0.80), and *non-hub kinless nodes* (*p*_*i*_ > 0.80), whereas hubs were classified into *provincial* (*p*_*i*_ ≤ 0.30), *connector* (0.30 < *p*_*i*_ ≤ 0.75), or *kinless hubs* (*p*_*i*_ > 0.75).

#### Intelligence-related differences in modular network organisation

All individual-differences analyses were done after exclusion of outliers, i.e., subjects with values > 3 *SD* above/below the mean of the respective variable of interest. For all whole-brain measures and proportions of node types we used SPSS22 (IBM Corp., Armonk, NY) to calculate partial correlations with WASI FSIQ, including potential confounding effects of age, sex, and handedness as covariates. Effects with *p* < 0.05 were considered statistically significant (Bonferroni-adjusted *p*-values are .013 for global modularity measures and .007 for node-type proportions). To quantify evidence for the null hypothesis (i.e., absence of an association) we calculated Bayes Factors^40,41^ (BF_01_), using Bayesian linear regression and the default prior^42^ as implemented in JASP (https://jasp-stats.org). In accordance with Jeffreys^40^, we interpret BF_01_ > 3 as substantial evidence for the null hypothesis.

To investigate the association between intelligence and whole-brain aspects of modular network organisation, we calculated partial correlations between WASI FSIQ and *global modularity Q*, *number of modules*, *average module size*, and the *variability in module size*. Furthermore, we tested for associations between intelligence and the whole-brain proportions of each node type as determined in the node-type analysis. To study the association between intelligence and node-specific aspects of modular organisation (i.e., between- and within-module connectivity), we set up two separate regression models in SPM8 (Statistic Parametric Mapping, Welcome Department of Imaging Neuroscience, London, UK), one for predicting the individual maps of *participation coefficient p*_*i*_, and one for predicting the individual maps of *within-module degree z*_*i*_ (both maps upsampled to 3 × 3 × 3 mm) by WASI FSIQ. To control for the potential confounding effects of age, sex, and handedness, these variables were included as covariates of no interest in all regression models. *P*-values were corrected for multiple comparisons using a Monte Carlo-based cluster-level thresholding procedure^43^. An overall threshold of *p* < 0.05 (FWE-corrected) was applied by combining a voxel-level threshold of *p* < 0.005 with a cluster-level threshold of *k* > 26 voxels (3dClustSim; AFNI version August 2016; 10,000 permutations; voxel size: 3 × 3 × 3 mm)^44^. As ultimately the modular organisation of the whole brain network is always defined by both, between-module and within-module connectivity, we also tested for an overlap of intelligence-related effects in both measures, i.e., *participation coefficient p*_*i*_ and *within-module degree z*_*i*_.

### Data availability statement

The data used in the present work can be accessed under the following link: http://fcon_1000.projects.nitrc.org/indi/enhanced/.

## Results

### Global modular organisation and intelligence

At the level of global brain network topology, we examined four measures of modular organisation, i.e., *global modularity Q*, *number of modules*, *average module size*, and *variability in module size*. Individual mean values for *global modularity Q* (averaged across the five different thresholds used to define the individual graphs) were all greater than 0.30 (*M* = 0.37; *SD* = 0.03), indicating a modular organisation of intrinsic brain networks in all subjects^34^. The individual average *numbers of modules* ranged from 2.80 to 4.80 (*M* = 3.54; *SD* = 0.33), while average *module sizes* ranged from 1149.01 to 2073.07 nodes (*M* = 1573.04, *SD* = 137.12). Finally, the individual average values for *variability in module size* ranged from 94.49 to 1105.95 nodes (*M* = 369.04; *SD* = 164.31), indicating that in some participants the brain network was partitioned into modules of nearly equal size, whereas in other participants the size of the modules differed substantially (for threshold-specific descriptive statistics for these measures, see Supplementary Table S1). None of the global measures of modular organisation was significantly associated with the WASI Full Scale Intelligence Quotient (Table 1; see Supplementary Table S2 for threshold-specific associations). These findings, resulting from the analysis of binary graphs, were replicated in the analysis of weighted graphs, where also no associations were observed between intelligence and the whole-brain measures of modular organisation (Supplementary Table S4).

**Table 1:** Associations between intelligence and whole-brain aspects of modular organisation.

	*r*_*part*._	*p*_*part*._	BF_01_-Reg.
*Whole-brain measures*			
global modularity	0.03	0.569	3.16
number of modules	0.04	0.531	3.07
average module size	−0.04	0.466	2.86
variability in module size	0.05	0.355	2.50
*Whole-brain proportions of node types*			
ultra-peripheral nodes	0.06	0.318	2.34
peripheral nodes	0.11	0.064	0.76
non-hub connector nodes	−0.03	0.528	3.07
non-hub kinless nodes	−0.04	0.462	2.86
provincial hubs	0.04	0.490	2.96
connector hubs	−0.03	0.586	3.21
kinless hubs	−0.03	0.657	3.35

*r*_*part*._, Pearson’s correlation coefficient for the partial correlation controlling for effects of age, sex, and handedness; *p*_*part*._, *p*-value of significance for the partial-correlation; BF_01_-Reg., Bayes Factor in favour of the null hypothesis (i.e., absence of correlation) for Bayes linear regression models predicting FSIQ values by the respective whole-brain measure of modular network organisation or whole-brain proportions of node types while controlling for effects of age, sex, and handedness.

We also assessed whether the global proportions of different node types (Fig. 2) were related to intelligence, as specific types of nodes (e.g., *connector hubs*, *provincial hubs*) may have a particularly strong impact on the information flow between and within modules^38,39^. Across participants, we observed the following distribution of node types: 1.80% (*SD* = 3.00%) *ultra-peripheral nodes*, 47.58% (*SD* = 7.84%) *peripheral nodes*, 22.79% (*SD* = 7.58%) *non-hub connector nodes*, 9.94% (*SD* = 3.51%) *non-hub kinless nodes*, 7.95% (*SD* = 3.30%) *provincial hubs*, 9.26% (*SD* = 3.44%) *connector hubs*, and 0.66% (*SD* = 0.59%) *kinless hubs* (Fig. 2B). For exemplary illustration, Fig. 2C visualises the anatomical distribution of node types for one single subject. These whole-brain proportions of node types were, however, also not associated with the WASI FSIQ (Table 1; see Supplementary Table S3 for threshold-specific associations). This was also true for weighted graphs (Supplementary Table S4).

### Node-specific aspects of modular organisation and intelligence

The node-specific analysis characterised each node by two measures: *participation coefficient p*_*i*_ (representing between-module connectivity) and *within-module degree z*_*i*_ (representing within-module connectivity). Across participants, a high *participation coefficient p*_*i*_ was observed for nodes in medial prefrontal cortex (mPFC), comprising anterior and mid-cingulate cortex (ACC, MCC), inferior frontal gyrus (IFG), anterior insula (AI), superior temporal gyrus (STG), inferior parietal lobule (IPL), posterior cingulate cortex (PCC)/precuneus, medial temporal structures (hippocampus, amygdala), and subcortically in the thalamus. High *within-module degree z*_*i*_ was observed for nodes in large parts of mPFC, also comprising ACC and MCC, lateral parts of superior and middle frontal gyrus (SFG, MFG), supplementary motor area (SMA), AI, postcentral gyrus, PCC/precuneus, temporo-parietal junction (TPJ), middle occipital/lingual gyrus (MOG/LG), and cuneus (Fig. 1).

**Figure 3:**
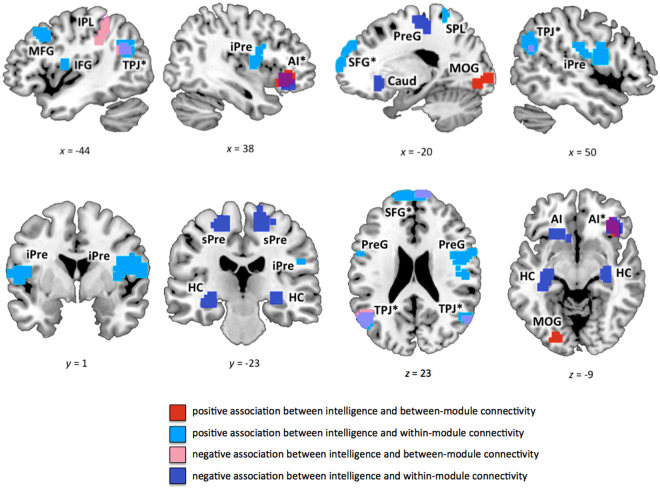
Clusters of nodes where intelligence was significantly associated with between-module or within-module connectivity (see also Table 2A,B). Between-module connectivity was operationalised by *participation coefficient p*_*i*_, within-module connectivity by *within-module degree z*_*i*_ (see Methods for more details). Graph metrics (*p*_*i*_ and *z*_*i*_) were calculated for binarised and proportionally thresholded graphs using five different cut-offs (i.e., graphs were defined by the 10%, 15%, 20%, 25%, or 30% strongest edges). For each participant, individual mean maps for the graph metrics were calculated by averaging across these five thresholds. Statistic parametric maps for both measures are shown at a voxel-level threshold of *p* < 0.005, uncorrected, combined with a cluster-level threshold of *k* > 26 voxels, corresponding to an overall threshold of *p* < 0.05, family-wise corrected for multiple comparisons (see Methods). Clusters with effects in both measures are marked with an asterisk (see also Fig. 4). SFG, superior frontal gyrus; AI, anterior insula; MFG, middle frontal gyrus; IFG, inferior frontal gyrus; SMA, supplementary motor area; Caud, caudate nucleus; HC, Hippocampus; iPre, inferior precentral gyrus; sPre, superior precentral gyrus; STG, superior temporal gyrus; TPJ, temporo-parietal junction; SPL, superior parietal lobule; IPL, inferior parietal lobule; MOG, middle occipital gyrus. The *x*-, *y*-, and *z*-coordinates represent coordinates of the Montreal Neurological Institute template brain (MNI152).

Several clusters of nodes in frontal and parietal cortex, but also in other cortical and subcortical structures, showed significant associations of between-module connectivity and within-module connectivity with intelligence (Table 2A,B; Fig. 3; for threshold-specific effects, see Supplementary Figure S1). WASI FSIQ scores were positively associated with *participation coefficient p*_*i*_ (between-module connectivity) in node clusters located in bilateral AI and left MOG/LG, whereas a negative association was observed for node clusters in medial SFG, left IPL, and bilateral TJP. Furthermore, WASI FSIQ scores were positively associated with *within-module degree z*_*i*_ (within-module connectivity) in node clusters in frontal cortex (medial SFG, left MFG, left precentral gyrus, right IFG) and parietal cortex (left SPL, bilateral TPJ), and negatively associated with node clusters in right AI, bilateral precentral gyrus, bilateral hippocampi, and subcortically in the left caudate nucleus. Most of these effects were replicated in the analysis for weighted graphs (with the exception of left AI and right TPJ for *p*_*i*_; see Supplementary Table S5).

**Table 2:** Intelligence-related effects in within-module and between-module connectivity.

Brain Region	BA	Hem	x	y	z	*t*_*max*_	*k*
A: *Participation coefficient p*_*i*_ (between-module connectivity)							
*positive association*							
anterior insula*	47, 13	R	36	33	−6	4.09	63
anterior insula	47, 13	L	−33	30	6	3.36	33
middle occipital / lingual gyrus	17, 18	L	−21	−84	−6	3.38	55
*negative association*							
superior frontal gyrus*	10	R/L	15	66	21	−2.61	38
inferior parietal lobule	40	L	−36	−45	42	−2.60	42
temporo-parietal junction*	39, 40	L	−51	−51	30	−2.60	175
temporo-parietal junction*	39	R	57	−63	24	−2.63	43
B: *Within-module degree z*_*i*_ (within-module connectivity)							
*positive association*							
superior frontal gyrus*	10, 9	R/L	−15	54	36	3.93	287
superior frontal gyrus	10	R	12	36	42	3.83	29
middle frontal gyrus	9, 8	L	−45	21	42	3.99	52
inferior precentral gyrus / superior temporal gyrus	22, 44	L	−54	0	9	4.10	108
inferior frontal gyrus / inferior precentral gyrus	44, 13	R	51	3	15	4.31	175
superior parietal lobule	5, 7	L	−36	−42	66	3.36	42
temporo-parietal junction*	39	L	−54	−63	27	4.41	177
temporo-parietal junction*	39	R	57	−63	33	4.04	65
*negative Association*							
anterior insula*	47, 13	R	33	30	−15	−2.61	81
superior precentral gyrus	4, 3	L	−18	−15	66	−2.63	65
superior precentral gyrus/supplementary motor area	6	R	21	−24	57	−2.60	108
hippocampus		L	−27	−21	−6	−2.61	74
hippocampus		R	33	−27	−6	−2.61	32
caudate nucleus		L	−6	24	−3	−2.60	71
C: Conjunction between *participation coefficient p*_*i*_ and *within-module degree z*_*i*_							
superior frontal gyrus	10	R/L	3	66	24		38
anterior insula	47, 13	R	33	30	−12		49
temporo-parietal junction	39, 40	L	−57	−69	21		116
temporo-parietal junction	39, 40	R	51	−60	24		35

BA, approximate Brodmann’s area; Hem, hemisphere; L, left; R, right; regions with intelligence-related effects in both measures (between-module and within-module connectivity) are marked with an asterisk and separately listed in (C); coordinates refer to the Montreal Neurological Institute template brain (MNI152); *t*_*max*_, maximum *t* statistic in the cluster; *k*, cluster size in voxels of size 3 × 3 × 3 mm.

**Figure 4:**
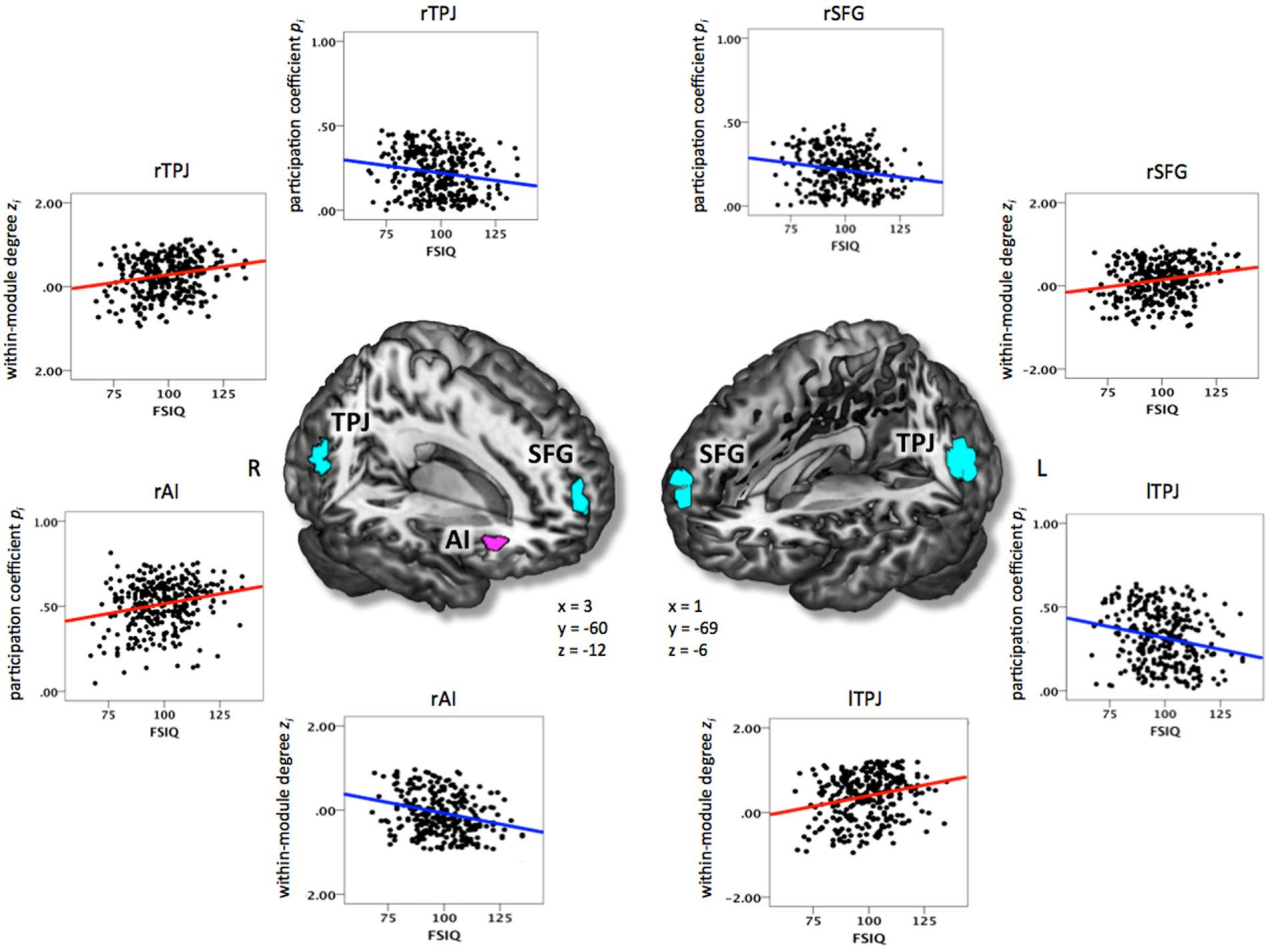
Clusters of nodes where intelligence was significantly associated with both modularity-defining dimensions (i.e., between-module and within-module connectivity; see also Table 2C). Between-module connectivity was operationalised by *participation coefficient p*_*i*_, within-module connectivity by *within-module degree z*_*i*_. Graph metrics (*p*_*i*_ and *z*_*i*_) were calculated for binarised and proportionally thresholded graphs using five different cut-offs (i.e., graphs were defined by the 10%, 15%, 20%, 25%, or 30% strongest edges). For each participant, individual mean maps for the graph metrics were calculated by averaging across the five thresholds. Statistic parametric maps for the conjunction between both measures are shown at a voxel-level threshold of *p* < 0.005, uncorrected, combined with a cluster-level threshold of *k* > 26 voxels, corresponding to an overall threshold of *p* < 0.05, corrected for multiple comparisons (see Methods). The scatterplots illustrate the associations between intelligence (i.e., WASI Full Scale Intelligence Quotient; FSIQ) and *participation coefficient p*_*i*_ as well as between intelligence and *within-module degree z*_*i*_ for the right superior frontal gyrus (rSFG), the right anterior insula (rAI), the left temporo-parietal junction area (lTPJ), and the right temporo-parietal junction (rTPJ). The *x*-, *y*- and *z*-coordinates represent coordinates of the Montreal Neurological Institute template brain (MNI152) and refer to the points of origin at which the slices were partially cut out. L, left; R, right.

As apparent in Fig. 3, four brain regions showed overlapping intelligence-related effects for *participation coefficient p*_*i*_ and *within-module degree z*_*i*_ (Table 2C; Fig. 4). In right AI, we observed a positive association for *participation coefficient p*_*i*_ and a negative association for *within-module degree z*_*i*_. Thus, in participants scoring higher on the WASI FSIQ, nodes in the right AI were characterised by higher connectivity to other modules along with lower connectivity within their own module. In medial SFG and bilateral TPJ, we observed the opposite pattern, i.e., WASI FSIQ was negatively associated with *participation coefficient p*_*i*_ and positively associated with *within-module degree z*_*i*_. Thus, in more intelligent subjects, nodes in SFG and TPJ were characterised by lower between-module and higher within-module connectivity.

## Discussion

Research from various fields, such as physics or computational biology, indicates several advantageous properties of modular network organisation, including adaptability, greater robustness^17^, or the minimisation of wiring costs^45^. Further, modularity has been suggested as a crucial precondition for functional specialisation^16^ – which is ubiquitous in the human brain.

In the current study, we investigated whether or not individual differences in the modular organisation of functional brain networks are associated with individual differences in the general capacity for higher cognition, i.e., intelligence. Intrinsic functional brain networks of all participants showed evidence of modular organisation^34^, and across participants, the distribution of the node-specific measures describing within- and between-module communication were consistent with those reported in previous studies^22,46,47^. General intelligence was associated with between- and within-module connectivity in node clusters located in frontal, parietal, and other cortical and subcortical brain regions that have previously been suggested as localised neural substrates of intelligence^3,4^. In contrast, topological properties of global modular network organisation were not associated with intelligence.

The fact that we observe intelligence-related differences in measures of modularity for node clusters in frontal, parietal, and other cortical and subcortical brain regions is well in line with the prevailing neurocognitive models of intelligence, i.e., the parieto-frontal integration theory (P-FIT)^3^ and its recent extension additionally considering subcortical structures^4^. However, while these models were derived from localisationist studies of morphological and brain-activation correlates of intelligence, we here provide converging evidence by adopting a distributed network perspective. Importantly, by explicitly taking into account the modular network structure of the brain, i.e., the fact that nodes are not uniformly distributed across the topology of the whole network but clustered into functionally partly independent modules^15^, we directly investigate correlates of segregated vs. integrated information processing. Early on, the P-FIT model^3,4^ has proposed that intelligence depends on the integration of information that is being processed in functionally specialised brain regions. Until today, however, empirical evidence in direct support of this proposal is still scarce, as for methodological reasons most previous studies could not inform about functional interactions between neural processing units, i.e., between functionally distinguishable brain modules. We propose that the specific embedding of intelligence-related brain regions, particularly involving frontal and parietal, but also other cortical and subcortical systems, provides advantages for information processing that are beneficial for higher cognitive performance.

### Segregated vs. integrated information processing

In general, modular organised networks are characterised by functional segregation and integration^48^. With respect to cognitive processing, it has been suggested that information processing within segregated modules may subserve specific cognitive functions, whereas the exchange of information between modules is accordingly assumed to be responsible for the coordination and integration of cognitive processes^46,49^. Broadly speaking, our results suggest that both contribute to intelligence. However, our results also indicate that intelligence depends on a differential and regionally specific tuning of these parameters. In AI and MOG, intelligence was positively associated with between-module connectivity, suggesting that in more intelligent subjects these regions show higher potential for the coordination of cognitive processes between different modules^46,49^. In other brain regions, we observed negative associations (IPL, SFG, TPJ), suggesting a possible role in shielding ongoing cognitive processing from interfering noise^19,50^. The finding of positive and negative associations is consistent with previous reports stating that both higher and lower levels of integration or segregation can be beneficial for cognitive performance^20^.

Within-module connectivity, in contrast, is typically interpreted as indication that a node or brain region contributes to information processing in support of a specific cognitive function^46,49^. High within-module connectivity may reflect that a node has a particularly strong influence on other nodes and/or is influenced by other nodes within the same module. Within-module connectivity also shows a differential pattern of positive and negative associations with intelligence, depending on the localisation of the specific node clusters. Successful cognitive performance in the sense of higher intelligence seems to be favoured by closer interactions of parieto-frontal brain regions (SFG, MFG, IFG, SPL, and TPJ) within their own module, whereas for other regions (AI, precentral gyrus, caudate, hippocampus) a more independent position within their own functional module seems to be beneficial.

### Opposing effects in between- and within-module connectivity

Beyond the simple associations with intelligence, four brain regions – right AI, medial SFG, and bilateral TPJ – exhibited overlapping and opposite associations between intelligence and within- vs. between-module connectivity. We speculate that these four brain regions may be special in the sense that cognitive performance (general intelligence) seems to benefit from an investment into one type of connectivity at the cost of the respective other. Thus, the AI (high *p*_*i*_, low *z*_*i*_) seems to be optimised for the integration and propagation of information across modules, while SFG and TPJ (high *z*_*i*_, low *p*_*i*_) seem to particularly strengthen information flow within distinct processing units. Functionally, these four brain regions have been linked to salience processing (AI)^51,52^ and the default mode of brain functioning (TPJ, SFG)^53–55^. It can be speculated that the higher between-module integration of AI in more intelligent persons may facilitate the coordinated processing of salient information between different modules, whereas the higher segregation of two key DMN regions in more intelligent persons may reduce the influence of potentially interfering information on goal-directed processing.

### No effects of global modularity on intelligence

Although significant associations between working memory performance and global network modularity have been reported in two previous studies^19,20^, and working memory is closely related to intelligence^23^, in our study, intelligence was neither associated with the global (whole-brain) measures of modular network organisation nor with individual differences in the whole-brain proportions of specific node types. However, the effects observed in the previous studies were of opposite direction (i.e., higher^19^ as well as lower^20^ *global modularity Q* for better performing subjects) and other investigations failed to replicate these findings^50,56^. Also in respect to node-type proportions, our null-findings seem to contradict initially reported positive associations between WM performance and proportions of connector hubs^20,22^. Potential explanations could be that both studies^20,22^ investigated individual differences in actual performance of n-back tasks, which may capture more specific and potentially more state-dependent differences in cognitive function, in contrast to rather stable individual differences in general intelligence and their associations with intrinsic properties of functional brain networks as investigated here.

### Nodal vs. global modularity

For the analysis of individual differences in graph models of human brain data, a discrepancy between findings at the node-specific and the whole-brain level is not uncommon^6,21,57^. Particularly when positive and negative effects are observed in node-specific measures (as in the current study), these can (at least partially) level out and thus do not have to impact the graph measures at the global level. Even though alterations in whole-brain modular organisation have been observed in several diseases like for example reduced modularity in autism^58^, or a smaller number of modules in Alzheimer’s disease^59^, it is still a matter of debate whether whole-brain aspects of modular organisation relate to individual differences in cognitive abilities in the unimpaired brain, and empirical evidence is rather heterogeneous^19–21^. One speculative conclusion that could be drawn from these results is that global differences in modular network organisation might be more pronounced in persons with pathologically impaired cognitive functions (and significantly altered brain networks) relative to healthy adult subjects, while such differences may be much smaller and without significant effect on cognitive ability within a healthy adult population. This speculation, however, requires further investigation.

### Limitations

We examined intelligence-related differences in network topology based on resting-state fMRI, i.e., when participants did not face a specific cognitive challenge. Even though resting-state functional connectivity is generally considered to reflect fundamental properties of brain function^7^ and recent research suggests a strong link between resting-state and task-related functional connectivity^9^, it remains a question for future research whether the observed intelligence-related differences in the intrinsic modular organisation of the brain persist in the presence of cognitive demands. Note that all analyses were based on individual module partitions. Theoretically, it may also be interesting to use a standard partition for all subjects, e.g., the 7-network partition provided by Yeo and colleagues^60^. However, as our study was specifically aimed at studying intelligence-related differences in the brain’s module structures, we deemed it essential to allow for individual differences at the level of module partitions. Future studies must show if and how individual differences in connectivity within and between modules depends on the specific partition chosen to represent the brain network’s community structure. Furthermore, the modularity maximisation approach we used to determine the participants’ individual module partitions is subject to a resolution limit that may prevent it from detecting smaller modules - which would possibly represent the network’s true module structure even better^34^. However, although the maximisation of global modularity *Q* is well established for the detection of brain modules^15^, and the Louvain algorithm is one of the most-widely used methods^20,35,58^, the average number of modules detected in our study is lower than has been reported for other resting-state network partitions^18,60^. Future studies will have to investigate in detail how the number of modules and in particular individual-difference effects in respective graph measures, may depend on various methodological choices (e.g., method used for community detection, node-parcellation scheme, thresholding, group-average vs. individual module partitions).

## Conclusion

In sum, the current study contributes to a network-based understanding of the biological basis of human intelligence in suggesting that region-specific profiles of intrinsic functional connectivity within and between different brain modules are relevant for individual differences in general cognitive ability. Although our results do not allow for causal inferences, our findings lend support to the idea that the *integration* of processing between functionally specialised brain regions plays an important role for intelligence. Though this idea has long been introduced theoretically in the P-FIT model of intelligence^3^, empirical evidence is still scarce. Our study goes beyond previous investigations in conceptualising the brain as a modular network and explicitly addressing the interactions between and within these modules. The specific topological embedding of intelligence-related network nodes, most of which were located in frontal and parietal cortex, may shape intelligence-relevant aspects of information processing. Specifically, this may reflect the facilitation of specific cognitive processes within segregated modules (e.g., SFG, TPJ) or the efficient integration of information throughout the brain (e.g., via the AI) in more intelligent persons. Understanding how differences in the brain’s modular organisation impact information processing provides an important avenue for understanding the neurobiological mechanisms underlying cognitive ability and general intelligence. The application of graph theory to the study of human brain imaging data provides the means for this endeavour.

## Electronic supplementary material

Supplementary Material

## References

[1] Gottfredson LS (1997). Mainstream science on intelligence: An editorial with 52 signatories, history, and bibliography. Intelligence.

[2] Gottfredson LS, Deary IJ (2004). Intelligence Predicts Health and Longevity, but Why?. Curr. Dir. Psychol. Sci..

[3] Jung, R. E. & Haier, R. J. The Parieto-Frontal Integration Theory (P-FIT) of intelligence: converging neuroimaging evidence. *Behav*. *Brain Sci*. **30**, 135–54; discussion 154–87 (2007). 10.1017/S0140525X07001185 17655784

[4] Basten U, Hilger K, Fiebach CJ (2015). Where smart brains are different: A quantitative meta-analysis of functional and structural brain imaging studies on intelligence. Intelligence.

[5] van den Heuvel MP, Stam CJ, Kahn RS, Hulshoff Pol HE (2009). Efficiency of functional brain networks and intellectual performance. J. Neurosci..

[6] Hilger K, Ekman M, Fiebach CJ, Basten U (2017). Efficient hubs in the intelligent brain: Nodal efficiency of hub regions in the salience network is associated with general intelligence. Intelligence.

[7] Biswal B, Yetkin FZ, Haughton VM, Hyde JS (1995). Functional connectivity in the motor cortex of resting human brain using echo-planar MRI. Magn. Reson. Med..

[8] Greicius MD, Supekar K, Menon V, Dougherty RF (2009). Resting-state functional connectivity reflects structural connectivity in the default mode network. Cereb. Cortex.

[9] Cole MW, Bassett DS, Power JD, Braver TS, Petersen SE (2014). Intrinsic and task-evoked network architectures of the human brain. Neuron.

[10] Cole MW, Ito T, Bassett DS, Schultz DH (2016). Activity flow over resting-state networks shapes cognitive task activations. Nat. Neurosci..

[11] Adelstein JS (2011). Personality is reflected in the brain’s intrinsic functional architecture. PLoS One.

[12] Menon V (2011). Large-scale brain networks and psychopathology: a unifying triple network model. Trends Cogn. Sci..

[13] Rubinov M, Sporns O (2010). Complex network measures of brain connectivity: uses and interpretations. Neuroimage.

[14] Ekman M, Derrfuss J, Tittgemeyer M, Fiebach CJ (2012). Predicting errors from reconfiguration patterns in human brain networks. PNAS.

[15] Sporns O, Betzel RF (2016). Modular Brain Networks. Annu. Rev. Psychol..

[16] Espinosa-Soto, C. & Wagner, A. Specialization can drive the evolution of modularity. *PLoS Comput*. *Biol*. **6** (2010). 10.1371/journal.pcbi.1000719 PMC2847948 20360969

[17] Kirschner M, Gerhart J (1998). Evolvability. PNAS.

[18] Sporns O (2014). Contributions and challenges for network models in cognitive neuroscience. Nat. Neurosci..

[19] Stevens AA, Tappon SC, Garg A, Fair DA (2012). Functional brain network modularity captures inter- and intra-individual variation in working memory capacity. PLoS One.

[20] Cohen JR, D’Esposito M (2016). The Segregation and Integration of Distinct Brain Networks and Their Relationship to Cognition. J. Neurosci..

[21] Liang X, Zou Q, He Y, Yang Y (2016). Topologically Reorganized Connectivity Architecture of Default-Mode, Executive-Control, and Salience Networks across Working Memory Task Loads. Cerebral Cortex.

[22] Stanley ML, Dagenbach D, Lyday RG, Burdette JH, Laurienti PJ (2014). Changes in global and regional modularity associated with increasing working memory load. Front. Hum. Neurosci..

[23] Kane, M. J., Hambrick, D. Z. & Conway, A. R. A. Working memory capacity and fluid intelligence are strongly related constructs: Comment on Ackerman, Beier, and Boyle (2005). *Psychol*. *Bull*. **131**, 66–71 (2005). 10.1037/0033-2909.131.1.66 15631552

[24] Nooner KB (2012). The NKI-Rockland Sample: A Model for Accelerating the Pace of Discovery Science in Psychiatry. Front. Neurosci..

[25] Wechsler, D. *Wechsler Abbreviated Scale of Intelligence* (Psychological Corporation, 1999).

[26] Ekman, M., & Linssen, C. Network-tools: Large-scale Brain Network Analysis in Python, 10.5281/zenodo.14803 (2015).

[27] Power JD (2011). Functional Network Organization of the Human Brain. Neuron.

[28] van Wijk, B. C. M., Stam, C. J. & Daffertshofer, A. Comparing brain networks of different size and connectivity density using graph theory. *PLoS One* **5**, (2010). 10.1371/journal.pone.0013701 PMC2965659 21060892

[29] Ginestet CE, Fournel AP, Simmons A (2014). Statistical network analysis for functional MRI: summary networks and group comparisons. Front. Comput. Neurosci..

[30] Bullmore ET, Bassett DS (2011). Brain graphs: graphical models of the human brain connectome. Annu. Rev. Clin. Psychol..

[31] Murphy K, Birn RM, Handwerker DA, Jones TB, Bandettini PA (2009). The impact of global signal regression on resting state correlations: Are anti-correlated networks introduced?. Neuroimage.

[32] Blondel VD, Guillaume J-L, Lambiotte R, Lefebvre E (2008). Fast unfolding of communities in large networks. J. Stat. Mech. Theory Exp..

[33] Newman MEJ, Girvan M (2004). Finding and evaluating community structure in networks. Phys. Rev. E.

[34] Fortunato S, Barthélemy M (2007). Resolution limit in community detection. PNAS.

[35] Meunier D, Lambiotte R, Fornito A, Ersche KD, Bullmore ET (2009). Hierarchical modularity in human brain functional networks. Front. Hum. Neurosci..

[36] Lancichinetti A, Fortunato S (2009). Community detection algorithms: A comparative analysis. Phys. Rev. E.

[37] Leicht EA, Newman MEJ (2008). Community structure in directed networks. Phys. Rev. Lett..

[38] Guimerà R, Amaral N (2005). Functional cartography of complex metabolic networks. Nature.

[39] Sporns R, Honey CJ, Kötter R (2011). Identification and Classification of Hubs in Brain Networks. Curr. Sci..

[40] Jeffreys, H. *Theory of probability*. (UK Oxford University Press, 1961).

[41] Wetzels R, Wagenmakers E-J (2012). A default Bayesian hypothesis test for correlations and partial correlations. Psychon. Bull. Rev..

[42] Rouder JN, Morey RD (2012). Default Bayes Factors for Model Selection in Regression. Multivariate Behav. Res..

[43] Forman SD (1995). Improved assessment of significant activation in functional magnetic resonance imaging (fMRI): use of a cluster-size threshold. Magn. Reson. Med..

[44] Ward, B. D. *Simultaneous inference for fMRI data**;* http://stuff.mit.edu/afs/sipb.mit.edu/project/seven/doc/AFNI/AlphaSim.ps (2000).

[45] Clune J, Mouret J-B, Lipson H (2013). The evolutionary origins of modularity. Proc. Biol. Sci..

[46] Gratton C, Nomura EM, Pérez F, D’Esposito M (2012). Focal brain lesions to critical locations cause widespread disruption of the modular organization of the brain. J. Cogn. Neurosci..

[47] Bertolero MA, Yeo BTT, D’Esposito M (2015). The modular and integrative functional architecture of the human brain. PNAS.

[48] Gallos LK, Makse HA, Sigman M (2012). A small world of weak ties provides optimal global integration of self-similar modules in functional brain networks. PNAS.

[49] Warren DE (2014). Network measures predict neuropsychological outcome after brain injury. PNAS.

[50] Gamboa OL (2014). Working memory performance of early MS patients correlates inversely with modularity increases in resting state functional connectivity networks. Neuroimage.

[51] Menon V, Uddin LQ (2010). Saliency, switching, attention and control: a network model of insula function. Brain Struct. Funct..

[52] Seeley WW (2007). Dissociable intrinsic connectivity networks for salience processing and executive control. J. Neurosci..

[53] Raichle ME (2001). A default mode of brain function. PNAS.

[54] Fox MD (2013). The human brain is intrinsically organized into dynamic, anticorrelated functional networks. PNAS.

[55] Basten U, Stelzel C, Fiebach CJ (2013). Intelligence is differentially related to neural effort in the task-positive and the task-negative brain network. Intelligence.

[56] Yeo RA (2016). Graph Metrics of Structural Brain Networks in Individuals with Schizophrenia and Healthy Controls: Group Differences, Relationships with Intelligence, and Genetics. J. Internat. Neuropsych. Society..

[57] Dennis EL (2013). Development of brain structural connectivity between ages 12 and 30: A 4-Tesla diffusion imaging study in 439 adolescents and adults. Neuroimage.

[58] Rudie JD (2013). Altered functional and structural brain network organization in autism. NeuroImage Clin..

[59] De Haan W (2012). Disrupted modular brain dynamics reflect cognitive dysfunction in Alzheimer’s disease. Neuroimage.

[60] Yeo BTT (2011). The organization of the human cerebral cortex estimated by intrinsic functional connectivity. J. Neurophysiol..

