# Supplementary material for: Intelligence is associated with the modular structure of intrinsic brain networks
Source: Sci Rep. 2017 Nov 22;7:16088. doi: 10.1038/s41598-017-15795-7 (PMC5700184; doi:10.1038/s41598-017-15795-7)
Supplement: Supplementary file 1 — Supplementary Material [file 41598_2017_15795_MOESM1_ESM.pdf]

## SUPPLEMENTARY MATERIAL

# Intelligence is associated with the modular structure of intrinsic brain networks

Kirsten Hilger[1,2,*], Matthias Ekman[3], Christian J. Fiebach[1,2,3], Ulrike Basten[1]

[1] Goethe University Frankfurt, Frankfurt am Main, Germany
[2] IDeA Center for Individual Development and Adaptive Education, Frankfurt am Main, Germany
[3] Donders Institute for Brain, Cognition, and Behaviour, Radboud University Nijmegen, The Netherlands

* Corresponding author:

Kirsten Hilger
Goethe University
Department of Psychology
Theodor-W.-Adorno-Platz 6, PEG
D-60323 Frankfurt am Main
Phone: +49 (0)69 / 798 - 35345
hilger@psych.uni-frankfurt.de

**Supplementary Table S1.** Descriptive statistics for global graph metrics at different graph thresholds.

| | *Thr* | *M* | *SD* | *Min* | *Max* |
|---|---|---|---|---|---|
| *Global modularity Q* | | | | | |
| | 10 % | .464 | . 037 | .258 | .578 |
| | 15 % | .408 | .034 | .208 | .532 |
| | 20 % | .365 | .034 | .207 | .497 |
| | 25 % | .330 | .035 | .201 | .462 |
| | 30 % | .300 | .034 | .212 | .441 |
| *Number of modules* | | | | | |
| | 10 % | 4.20 | .595 | 3 | 6 |
| | 15 % | 3.74 | .556 | 3 | 5 |
| | 20 % | 3.41 | .512 | 2 | 5 |
| | 25 % | 3.21 | .434 | 2 | 5 |
| | 30 % | 3.14 | .353 | 2 | 4 |
| *Module size* | | | | | |
| | 10 % | 1314.41 | 192.82 | 897.67 | 1803.67 |
| | 15 % | 1478.55 | 229.27 | 1075.00 | 1803.67 |
| | 20 % | 1620.88 | 232.83 | 1077.29 | 2704.00 |
| | 25 % | 1709.22 | 204.54 | 1078.60 | 2704.00 |
| | 30 % | 1742.16 | 165.25 | 1332.00 | 2672.50 |
| *Variability in module size* | | | | | |
| | 10 % | 376.96 | 183.08 | 34.00 | 932.69 |
| | 15 % | 368.27 | 183.06 | 27.64 | 1031.39 |
| | 20 % | 368.27 | 182.86 | 15.06 | 1138.56 |
| | 25 % | 360.02 | 223.71 | 13.82 | 1222.94 |
| | 30 % | 397.72 | 276.54 | 11.05 | 1300.46 |

*Thr*, proportional threshold applied on the binarised adjacency matrix; *M*, mean, *SD*, standard deviation; *Min*, minimum; *Max*, maximum.

**Supplementary Table S2.** Associations between intelligence (WASI FSIQ) and whole-brain aspects of modular organization across thresholds.

| | ***Thr*** | $r_{part.}$ | $p_{part.}$ | **$BF_{01}$-Reg.** |
|---|---|---|---|---|
| *Global Modularity Q* | | | | |
| | 10 % | .042 | .470 | 2.884 |
| | 15 % | .004 | .952 | 3.665 |
| | 20 % | .025 | .669 | 3.369 |
| | 25 % | .057 | .327 | 2.369 |
| | 30 % | .046 | .432 | 2.772 |
| *Number of modules* | | | | |
| | 10 % | .085 | .144 | 1.384 |
| | 15 % | .095 | .099 | 1.060 |
| | 20 % | -.045 | .441 | 2.803 |
| | 25 % | .041 | .480 | 2.914 |
| | 30 % | -.099 | .088 | 0.970 |
| *Module size* | | | | |
| | 10 % | -.089 | .122 | 1.226 |
| | 15 % | -.088 | .127 | 1.311 |
| | 20 % | .029 | .610 | 3.251 |
| | 25 % | -.063 | .277 | 2.113 |
| | 30 % | .099 | .088 | 0.971 |
| *Variability in module size* | | | | |
| | 10 % | .035 | .550 | 3.128 |
| | 15 % | .098 | .091 | 0.985 |
| | 20 % | -.014 | .813 | 3.592 |
| | 25 % | .005 | .926 | 3.635 |
| | 30 % | -.033 | .569 | 3.130 |

*Thr*, proportional threshold applied on the binarised adjacency matrix; $r_{part.}$, Pearson's correlation coefficient for the partial correlation controlling for effects of age, sex, and handedness; $p_{part.}$, *p*-value of significance for the partial-correlation; $BF_{01}$-Reg., Bayes Factor in favour of the null hypothesis (i.e., absence of correlation) for Bayes linear regression models predicting FSIQ values by the respective whole-brain measure of modular network organization while controlling for effects of age, sex, and handedness.

**Supplementary Table S3.** Associations between intelligence (WASI FSIQ) and whole-brain node type proportions at different graph thresholds.

| | ***Thr*** | $r_{part.}$ | $p_{part.}$ | **$BF_{01}$-Reg.** |
|---|---|---|---|---|
| *Ultra-peripheral nodes* | | | | |
| | 10 % | .030 | .610 | 3.238 |
| | 15 % | .015 | .801 | 3.557 |
| | 20 % | -.057 | .327 | 2.379 |
| | 25 % | -.028 | .632 | 3.275 |
| | 30 % | .021 | .723 | 3.499 |
| *Peripheral nodes* | | | | |
| | 10 % | .062 | .287 | 2.184 |
| | 15 % | .070 | .226 | 1.883 |
| | 20 % | -.050 | .390 | 2.621 |
| | 25 % | .062 | .284 | 2.177 |
| | 30 % | .102 | .078 | 0.890 |
| *Non-hub connector nodes* | | | | |
| | 10 % | -.020 | .728 | 3.491 |
| | 15 % | -.045 | .439 | 2.805 |
| | 20 % | -.013 | .819 | 3.613 |
| | 25 % | -.031 | .594 | 3.241 |
| | 30 % | -.027 | .642 | 3.342 |
| *Non-hub kinless nodes* | | | | |
| | 10 % | .023 | .694 | 3.438 |
| | 15 % | .062 | .280 | 2.161 |
| | 20 % | .104 | .071 | 0.832 |
| | 25 % | .009 | .882 | 3.647 |
| | 30 % | -.135 | .020* | 0.305 |
| *Provincial hubs* | | | | |
| | 10 % | .010 | .860 | 3.637 |
| | 15 % | .024 | .675 | 3.369 |

| | | | | |
|---|---|---|---|---|
| | 20 % | -.046 | .421 | 2.745 |
| | 25 % | .045 | .433 | 2.783 |
| | 30 % | .027 | .640 | 3.028 |
| *Connector hubs* | | | | |
| | 10 % | .011 | .852 | 3.631 |
| | 15 % | -.031 | .596 | 3.244 |
| | 20 % | -.040 | .489 | 2.963 |
| | 25 % | -.040 | .493 | 2.977 |
| | 30 % | -.015 | .796 | 3.578 |
| *Kinless hubs* | | | | |
| | 10 % | .001 | .991 | 3.672 |
| | 15 % | .058 | .322 | 2.342 |
| | 20 % | .068 | .239 | 1.956 |
| | 25 % | .032 | .578 | 3.186 |
| | 30 % | -.033 | .568 | 3.380 |

*Thr*, proportional threshold applied on the binarised adjacency matrix; $r_{part.}$, Pearson's correlation coefficient for the partial correlation controlling for effects of age, sex, and handedness; $p_{part.}$, *p*-value of significance for the partial-correlation; $BF_{01}$-Reg., Bayes Factor in favour of the null hypothesis (i.e., absence of correlation) for Bayes linear regression models predicting FSIQ values by the respective node-type proportion while controlling for effects of age, sex, and handedness.

**Supplementary Table S4**. Associations between intelligence (WASI FSIQ) and whole-brain aspects of modular organization based on weighted graphs.

| | $r_{part.}$ | $p_{part.}$ | **$BF_{01}$-Reg.** |
|---|---|---|---|
| *Whole-brain measures* | | | |
| global modularity | .03 | .632 | 3.31 |
| number of modules | .10 | .094 | 1.02 |
| average module size | -.10 | .094 | 1.19 |
| variability in module size | .08 | .170 | 1.55 |
| *Whole-brain proportions of node types* | | | |
| ultra-peripheral nodes | .09 | .145 | 1.34 |
| peripheral nodes | .05 | .420 | 2.74 |
| non-hub connector nodes | -.03 | .616 | 3.29 |
| non-hub kinless nodes | .04 | .521 | 3.06 |
| provincial hubs | .04 | .518 | 3.05 |
| connector hubs | -.03 | .616 | 3.29 |
| kinless hubs | .03 | .672 | 3.40 |

$r_{part.}$, Pearson's correlation coefficient for the partial correlation controlling for effects of age, sex, and handedness; $p_{part.}$, $p$-value of significance for the partial-correlation; $BF_{01}$-Reg., Bayes Factor in favour of the null hypothesis (i.e., absence of correlation) for Bayes linear regression models predicting FSIQ values by the respective whole-brain measure of modular network organization or whole-brain proportions of node types while controlling for effects of age, sex, and handedness.

**Supplementary Table S5.** Intelligence-related effects in weighted within-module and between-module connectivity.

| Brain Region | BA | Hem | x | y | z | $t_{max}$ | *k* |
|---|---|---|---|---|---|---|---|
| A: Weighted *participation coefficient* $p_{iw}$ (between-module connectivity) | | | | | | | |
| *positive association* | | | | | | | |
| anterior insula* | 47, 13 | R | 36 | 33 | -6 | 4.03 | 58 |
| cuneus | 30, 23 | L | -9 | -72 | 6 | 3.36 | 32 |
| middle occipital gyrus | 17, 18 | L | -18 | -93 | -3 | 3.65 | 130 |
| *negative association* | | | | | | | |
| superior frontal gyrus* | 10 | R/L | 3 | 63 | 12 | -2.62 | 61 |
| inferior parietal lobule | 40 | L | -42 | -39 | 39 | -2.60 | 28 |
| temporo-parietal junction* | 39, 40 | L | -48 | -66 | 30 | -2.60 | 136 |
| B: Weighted *within-module degree* $z_{iw}$ (within-module connectivity) | | | | | | | |
| *positive association* | | | | | | | |
| superior frontal gyrus* | 10, 9 | R/L | -15 | 54 | 36 | 3.97 | 300 |
| superior frontal gyrus | 8 | L | -12 | 33 | 51 | 3.23 | 31 |
| middle frontal gyrus | 9, 8 | L | -45 | 21 | 42 | 3.94 | 61 |
| inferior precentral gyrus / superior temporal gyrus | 22, 44 | L | -54 | 0 | 6 | 3.49 | 34 |
| inferior frontal gyrus / inferior precentral gyrus | 44, 13 | R | 45 | 3 | 21 | 3.43 | 40 |
| superior parietal lobule | 5, 7 | L | -27 | -48 | 69 | 3.04 | 35 |
| temporo-parietal junction* | 39 | L | -51 | -66 | 27 | 4.35 | 217 |
| temporo-parietal junction | 39 | R | 57 | -63 | 33 | 4.28 | 67 |
| *negative association* | | | | | | | |
| anterior insula* | 47, 13 | R | 36 | 30 | -3 | -2.56 | 75 |
| superior precentral gyrus | 4, 3 | L | -15 | -18 | 63 | -2.59 | 80 |
| superior precentral gyrus / supplementary motor area | 6, 4 | R | 39 | -18 | 54 | -2.61 | 139 |

| | | | | | | | |
|---|---|---|---|---|---|---|---|
| hippocampus | | L | -33 | -27 | -12 | -2.60 | 73 |
| hippocampus | | R | 27 | -15 | -9 | -2.60 | 36 |
| caudate nucleus | | L | -9 | 27 | 0 | -2.65 | 52 |
| C: Conjunction between weighted *participation coefficient* $p_{iw}$ and *weighted within-module degree* $z_{iw}$ | | | | | | | |
| superior frontal gyrus | 10 | R/L | 3 | 63 | 12 | | 61 |
| anterior insula | 47, 13 | R | 33 | 27 | -9 | | 41 |
| temporo-parietal junction | 39, 40 | L | -57 | -69 | 21 | | 98 |

BA, approximate Brodmann's area; Hem, hemisphere; L, left; R, right; regions with intelligence-related effects in both measures (between-module and within-module connectivity) are marked with an asterisk and separately listed in (C); coordinates refer to the Montreal Neurological Institute template brain (MNI); $t_{max}$, maximum $t$ statistic in the cluster; $k$, cluster size in voxels of size 3x3x3mm.

**Supplementary Figure 1.** Clusters of nodes where intelligence (WASI FSIQ) was significantly associated with between-module or within-module connectivity for each of 5 different graph threshold.

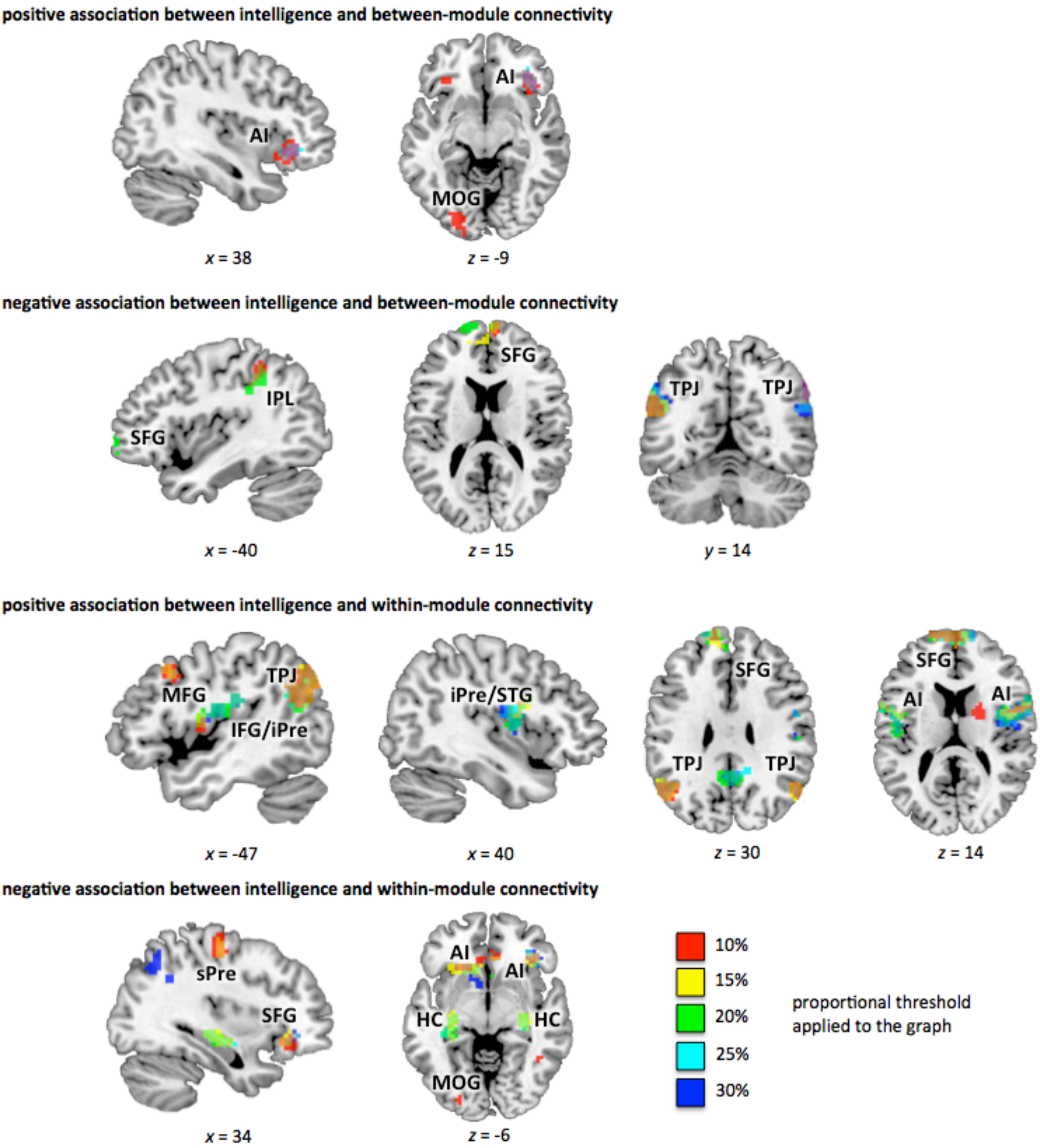


Between-module connectivity was operationalized by *participation coefficient* $p_i$ , within-module connectivity by *within-module degree* $z_i$ (see Methods for details). Statistic parametric maps for both measures are shown at a voxel-level threshold of $p < .005$, uncorrected, and a cluster-level threshold of $k > 26$ voxels, corresponding to an overall threshold of $p < .05$, family-wise corrected for multiple comparisons (see Methods). SFG, superior frontal gyrus; AI, anterior insula; MFG, middle frontal gyrus; IFG, inferior frontal gyrus; HC, Hippocampus; iPre, inferior precentral gyrus; sPre, superior precentral gyrus; STG, superior temporal gyrus; TPJ, temporo-parietal junction; SPL, superior parietal lobule; IPL, inferior parietal lobule; MOG, middle occipital gyrus. The *x*-, *y*- and *z*-coordinates represent coordinates of the Montreal Neurological Institute template brain (MNI152).
